# Effect of Neuromuscular Electrical Stimulation Training on the Finger Extensor Muscles for the Contralateral Corticospinal Tract in Normal Subjects: A Diffusion Tensor Tractography Study

**DOI:** 10.3389/fnhum.2018.00432

**Published:** 2018-11-20

**Authors:** Sung Ho Jang, You Sung Seo

**Affiliations:** Department of Physical Medicine and Rehabilitation, College of Medicine, Yeungnam University, Daegu, South Korea

**Keywords:** neuromuscular electrical stimulation, diffusion tensor tractography, corticospinal tract, hand function, finger extensor

## Abstract

**Objectives**: Neuromuscular electrical stimulation (NMES) is a popular rehabilitative modality to improve motor function of the extremities and trunk. In this study, we investigated changes of hand function and the contralateral corticospinal tract (CST) with treatment by NMES on the finger extensor muscles for 2 weeks, using serial diffusion tensor tractography (DTT).

**Methods**: Thirteen right handed normal subjects were recruited. Treatment was applied to the left hand (the NMES side), and the right hand was the control side. NMES was applied for 30 min/day, 7 days per week, for 2 weeks. Hand motor function was evaluated twice at pre-NMES and post-NMES training using grip strength (GS), Purdue pegboard test (PPT) and tip pinch. The fractional anisotropy (FA), mean diffusivity (MD) and tract volume (TV) of the CST in both hemispheres were measured using DTT.

**Results**: On the control side, the clinical scores did not differ significantly between pre- and post-NMES training (*p* > 0.05). However, on the NMES side, PPT and tip pinch improved significantly (*p* < 0.05), although GS did not. TV of the right CST increased significantly at post-NMES training (*p* < 0.05) whereas FA and MD did not differ significantly (*p* > 0.05). By contrast, FA, MD and TV on the left CST did not change significantly (*p* > 0.05).

**Conclusion**: We demonstrated facilitation of the contralateral CST with improvement of fine motor activity by 2 weeks of NMES training of peripheral muscles in normal subjects. We think our results can be applied to the normal subjects and patients with brain injury to improve the fine motor function of the hand and facilitate the normal CST or healing of the injured CST.

## Introduction

Neuromuscular electrical stimulation (NMES), a popular rehabilitative modality, induces contraction of neuromuscular system by applying electrical current (Rushton, 1997; Powell et al., 1999; Chae and Yu, 2000; Daly and Ruff, 2007; Kim et al., 2010; Doucet et al., 2012; Maddocks et al., 2013; de Oliveira Melo et al., 2013). In the field of rehabilitation, NMES has long been used to improve motor function of the muscles of extremities and trunk, and the working mechanisms have been suggested as improvement of muscle strength, decrease of spasticity of antagonist muscles, increased range of motion, improvement of voluntary motor control and recovery of functional movement (Rushton, 1997; Powell et al., 1999; Chae and Yu, 2000; Daly and Ruff, 2007; Kim et al., 2010; Doucet et al., 2012; Maddocks et al., 2013; de Oliveira Melo et al., 2013). Furthermore, several studies have reported that NMES facilitates healing of the corticospinal tract (CST) directly (Han et al., 2003; Mang et al., 2010, 2011; Wei et al., 2013; Chen et al., 2014).

The CST is the most important neural tract for motor function in the human brain, and is associated with voluntary movements of proximal and distal musculature, especially fine motor activity of the hand (York, 1987; Davidoff, 1990; Jang, 2014; Jang et al., 2014b). To improve motor function, it is important to facilitate the CST in both normal subjects and patients with brain injury. Many studies have demonstrated CST healing using repetitive transcranial magnetic stimulation (rTMS), hand-arm bimanual intensive therapy or NMES (Han et al., 2003; Kim et al., 2006; Khedr et al., 2010; Mang et al., 2010, 2011; Wei et al., 2013; Chen et al., 2014; Weinstein et al., 2015; Chang et al., 2016). These studies evaluated their effect using functional magnetic resonance imaging (fMRI) and diffusion tensor imaging (DTI), although these methods have limited precision to evaluate change of the entire CST (Han et al., 2003; Kim et al., 2006; Khedr et al., 2010; Mang et al., 2010, 2011; Wei et al., 2013; Chen et al., 2014; Weinstein et al., 2015; Chang et al., 2016).

DTI has a unique advantage in identification and estimation of subcortical white matter by virtue of their ability to visualize water diffusion characteristics. However, it is difficult to get objective results because the results could be subjective depending on the location of the region of interest (ROI) which is applied by a data analyzer. By contrast, diffusion tensor tractography (DTT) for reconstruction of the neural tracts usually employs a combined ROI method that reconstructs only neural fibers passing more than two ROI areas. The ROI areas and reconstruction conditions for the neural tracts are well-defined for each neural tract (Mori et al., 1999; Wakana et al., 2007; Malykhin et al., 2008; Wang et al., 2012; Lee and Jang, 2015; Brandstack et al., 2016; Jang, 2016). High repeatability and reliability of DTT method for the neural tracts have been demonstrated in many studies (Mori et al., 1999; Wakana et al., 2007; Malykhin et al., 2008; Danielian et al., 2010; Wang et al., 2012; Seo and Jang, 2014; Lee and Jang, 2015; Brandstack et al., 2016; Jang, 2016). Therefore, experienced analyzers can reconstruct the neural tracts without significant inter- and intra-analyzer variation. The main advantage of DTT over DTI is that the entire neural tract can be evaluated in terms of DTT parameters, including fractional anisotropy (FA), mean diffusivity (MD) and tract volume (TV) and configurational analysis. DTT enables three-dimensional reconstruction and estimation of the CST in the human brain (Mori et al., 1999; Yamada et al., 2003; Puig et al., 2010). Therefore, we think that DTT would be more appropriate than fMRI or DTI to detect change of the entire CST. We hypothesized that application of the NMES on the finger extensor muscles could facilitate the contralateral CST that can be evaluated precisely with serial DTTs.

In the current study, we investigated changes of hand function and the contralateral CST with application NMES on the finger extensor muscles for 2 weeks, using serial DTTs.

## Materials and Methods

### Subjects

Thirteen right-handed healthy subjects (five males, eight females; 23.23 ± 3.59, range 21–33) were recruited according to the following criteria: (1) no previous history of psychiatric, neurological, or physical illness; (2) no brain lesion on conventional MRI, confirmed by a neuroradiologist; and (3) right handed, confirmed by the Edinburgh Handedness Inventory (Oldfield, 1971). Treatment was applied to the left hand (the NMES side), and the right hand was the control side. All subjects provided written informed consent prior to the start of the study, and the study protocol was approved by the Institutional Review Board of a Yeungnam University hospital.

### Neuromuscular Electrical Stimulation (NMES) Training

NMES was applied through a two-channel electrical simulator (EMGFES 1,000, Cyber Medic, South Korea). Monophasic square wave pulses were used at the rate of 30 Hz with a pulse width of 200 μs, pulsed 3 s on and 2 s off. Square surface stimulation electrodes were used to activate finger extensor muscles of the left hand (fixed to the skin with adhesive gel). The electrodes were positioned with a cathode over the left extensor digitorum communis and an anode on the left forearm near the wrist. The stimulation intensity was adjusted to produce the maximum extension of the finger within the limit that the subject did not feel any discomfort (range of stimulation intensity: 8 ~ 13 mA; Shin et al., 2008; Jang et al., 2014a). The subjects were given NMES training as follows: 30 min/day, 7 days per week for 2 weeks.

### Clinical Evaluation

Grip strength (GS), Purdue pegboard test (PPT) and tip pinch were used evaluate hand function at pre- and post-NMES training. There are several clinical evaluation tools for these parameters. For GS, the subjects were asked to sit on a chair with their hip joint flexed at 90°, and shoulder joint in a neutral position, elbow fixed at 90° flexion, forearm in a neutral position, and wrist at 0° to 15° radial deviation. The Jamar dynamometer (Jamar Hydraulic Hand Dynamometer, model-5030J1) was used to evaluate GS. For PPT (Lafayette instruments, model 32020), the subjects were required to place as many pegs as possible in 30-s periods using the right hand and left hand. For tip pinch, the subjects push the tip of index finger and hold the pinch gauge with thumb. A hydraulic pinch gauge measured the force between index finger and thumb, parameters indicate the strength of the two fingers (Tiffin and Asher, 1948; Smith and Benge, 1985; Reddon et al., 1988; Kim et al., 1994; Kong et al., 2014). All of the clinical evaluations were performed three times and average value was calculated.

### Fiber Tracking

Using a six-channel head coil with single-shot echo planar imaging on 1.5 T (Philips Ltd., Best, Netherlands), DTI was acquired at pre- and post-NMES training (2 weeks after pre-NMES-training). For each of the 32 non-collinear diffusion sensitizing gradients, 70 contiguous slices (number of excitations: 1, imaging reduction factor (sensitivity encoding (SENSE) factor): 2, field of view: 240 × 240 mm^2^, reconstructed to matrix: 192 × 192, acquisition matrix: 96 × 96, parallel echo planar imaging factor: 59, TE: 72 ms, TR: 10, 398 ms, b: 1,000 s/mm^2^, and a slice thickness of 2.5 mm) were acquired. To analyze the CST, the single-tensor model was used within the DTI task card software (Philips Extended MR Workspace 2.6.3). Each DTI replication was intra-registered to the baseline “b0” images to correct for residual eddy-current image distortions and head motion effect, using a diffusion registration package (Philips Medical Systems). DTI-Studio software (CMRM, Johns Hopkins Medical Institute, Baltimore, MD, USA) was used for reconstruction of the CST. DTI-Studio is one of the most popular and commonly used the program for analysis of DTI data. Furthermore, it has an advantage of applying to the various sources of data and the efficient fiber tracking. In detail, for the fiber tracking, two thresholds (FA and tract turning-angle) was used and tracking is performed from all pixels, in which FA values and turning-angle are higher and lower than thresholds. Fiber tracking was based on the fiber assignment continuous tracking algorithm and a multiple ROIs approach. For reconstruction of the CST, ROI was placed on the upper pons (portion of anterior blue color) on the color map with an axial image. The second ROI was placed on the mid pons (portion of anterior blue color) on the color map with an axial image. The termination criteria used default value FA <0.2, angle <60° (Kunimatsu et al., 2004).

### Statistical Analysis

SPSS software (SPSS Inc. Released 2006. SPSS for Windows, Version 15.0. Chicago) was used for data analysis. The paired *t*-test was used for determination of differences in values of clinical scores and DTT parameters of the subjects between the NMES and control sides. Pearson correlation coefficients were calculated to assess the strength of association between clinical scores (GS, PPT and tip pinch) and DTT parameters of the CST. Null hypotheses of no difference were rejected if *p*-values were less than 0.05.

## Results

Table 1 shows average scores of GS, PPT and tip pinch between the NMES and control sides in pre- and post-NMES training. On the control side, no clinical scores (GS, PPT and tip pinch) differed significantly between pre- and post-NMES training (*p* > 0.05). On the NMES side, PPT and tip pinch improved significantly (*p* < 0.05) with the NMES training, although GS did not.

**Table 1:** Clinical scores at pre- and post-neuromuscular electrical stimulation training.

		Pre-NMES training	Post-NMES training	*p*-value
GS	Control	32.1 ± 8.8	32.2 ± 7.6	0.127
	NMES	33.2 ± 9.1	33.2 ± 9.7	0.387
PPT	Control	14.8 ± 1.7	14.7 ± 1.9	0.144
	NMES	16.3 ± 1.9	16.8 ± 1.7	0.018*
Tip pinch	Control	3.2 ± 1.5	3.2 ± 1.8	0.377
	NMES	3.5 ± 1.3	3.8 ± 1.4	0.003*

NMES, neuromuscular electrical stimulation; GS, grip strength; PPT, Purdue pegboard test. Values mean ± standard deviation. *Significant differences between pre- and post-NMES trainings, p < 0.05.

**Figure 1:**
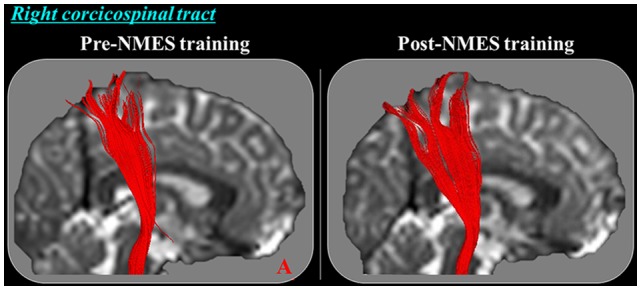
Diffusion tensor tractography (DTT) for the right corticospinal tract (CST) in a representative subject (21-year old female). The right CST on post-neuromuscular electrical stimulation (NMES) training become thicker compared with pre-NMES training in this subject.

**Figure 2:**
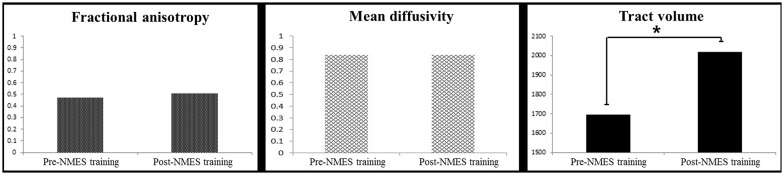
Comparison of group analysis of DTT parameters for the right CST of pre- and post-NMES training (*p < 0.05).

A summary of comparison of the DTT parameters between the right and left CSTs is shown Table 2. Regarding the configuration, the TV of the right CST shows more thicker at post-NMES training compared with pre-NMES training (Figure 1); and TV of the right CST increased significantly at post-NMES training compared with pre-NMES training (*p* < 0.05) whereas FA and MD did not change significantly (*p* > 0.05; Figure 2). In contrast, FA, MD and TV did not change in the left CST between pre-NMES and post-NMES trainings (*p* > 0.05).

**Table 2:** Diffusion tensor tractography (DTT) parameters at pre- and post-neuromuscular electrical stimulation training.

		Pre-NMES training	Post-NMES training	*p*-value
FA	NMES	0.47 ± 0.13	0.51 ± 0.02	0.374
	Control	0.51 ± 0.02	0.51 ± 0.03	0.391
MD	NMES	0.84 ± 0.05	0.84 ± 0.05	0.635
	Control	0.83 ± 0.05	0.83 ± 0.05	0.528
TV	NMES	1696.85 ± 559.88	2017.23 ± 490.48	0.001*
	Control	1857.23 ± 712.22	1986.62 ± 657.27	0.399

NMES, neuromuscular electrical stimulation; FA, fractional anisotropy; MD, mean diffusivity; TV, tract volume. Values: mean ± standard deviation *Significant differences between pre- and post-NMES trainings, p < 0.05.

Correlation coefficients did not differ significantly between clinical scores (GS, PPT and tip pinch) and DTT parameters (FA (GS: *r* = 0.263, *p* > 0.05; PPT: *r* = 0.325, *p* > 0.05; tip pinch: *r* = 0.442, *p* > 0.05), MD (GS: *r* = 0.342, *p* > 0.05; PPT: *r* = 0.441, *p* > 0.05 ; tip pinch: *r* = 0.612, *p* > 0.05) and TV (GS: *r* = 0.658, *p* > 0.05; PPT: *r* = 0.335, *p* > 0.05; tip pinch: *r* = 0.741, *p* > 0.05)) in the NMES and control sides (*p* > 0.05).

## Discussion

In the current study, using DTT, we investigated change of the CST between pre- and post-NMES training with application of NMES on the finger extensor muscles for 2 weeks. Our results can be summarized as follows. First, PPT and tip pinch improved on the NMES (left hand) side without change of GS. Second, TV of the right CST that innervates the left finger extensor muscles, increased after NMES training without change of FA and MD value.

In neuro-rehabilitaion, NMES is commonly applied to the finger extensor muscles of the affected hand because the affected hand usually shows flexor spasticity in hemiparetic patients with brain injury (Nakipğlu Yuzer et al., 2017; Pundik et al., 2018). Therefore, NMES was applied to the finger extensor muscles in this study. NMES training produced improvements in PPT and tip pinch, although not in GS. PPT and tip pinch represent finer motor function of hand than GS. This suggests that NMES treatment of the finger extensor muscles might be more effective in improving finer motor activity than gross muscle power. This appears consistent with studies showing that the CST is important in fine motor activity and strength (Kim et al., 2006; Khedr et al., 2010; Mang et al., 2010, 2011; Weinstein et al., 2015; Chang et al., 2016). In addition, NMES training which was applied to extend the finger extensors maximally appeared to facilitate the function of the finger extensors which are needed to perform finer motor function of the hand.

Among DTT parameters, FA, MD and TV have most commonly been used to evaluate the state of a neural tract (Assaf and Pasternak, 2008; Neil, 2008; Pagani et al., 2008). FA value indicates the degree of directionality of water diffusion and the white matter organization; in detail, the degree of directionality and integrity of white matter microstructures such as axon, myelin, and microtubule (Assaf and Pasternak, 2008; Neil, 2008; Pagani et al., 2008). The MD value indicates the magnitude of water diffusion in tissue, and TV is determined by the number of voxels included in a neural tract, thereby suggesting the total number of fibers of a neural tract (Pagani et al., 2008). Therefore, increased values of TV in the CST indicate increment in fiber number of the CST compared with post-NMES training (Pagani et al., 2008; Jang et al., 2013). This result agrees with studies that report association between improvement of motor function and increment of TV of the CST (Schaechter et al., 2009; Jang et al., 2014b; Seo and Jang, 2015).

Many studies have tried to facilitate healing of the CST using rehabilitative interventions including rTMS, hand-arm bimanual intensive therapy, and NMES (Han et al., 2003; Kim et al., 2006; Khedr et al., 2010; Mang et al., 2010, 2011; Wei et al., 2013; Chen et al., 2014; Weinstein et al., 2015; Chang et al., 2016). Several studies demonstrated an activating or facilitating effect of NMES on the CST (Kim et al., 2006; Khedr et al., 2010; Mang et al., 2010, 2011; Weinstein et al., 2015; Chang et al., 2016). In 2003, Han et al. reported activation of the primary motor cortex by application of NMES on the wrist extensor muscles in eight normal subjects using fMRI (Han et al., 2003). In 2010, Mang et al. demonstrated that applying 100 Hz on the common peroneal nerve is the most appropriate frequency of NMES to facilitate the CST in eight normal subjects using motor-evoked potential (MEP) on transcranial magnetic stimulation (TMS; Mang et al., 2010). The next year, Mang et al. (2011) studied the effect of NMES on target muscle for 40 min in 14 normal subjects and found the facilitation of the CST using MEP on TMS. Using DTI, two studies reported the facilitation of the CST using NMES (Wei et al., 2013; Chen et al., 2014). In 2013, Wei et al. investigated the effect of NMES on the wrist extensor muscles for 20 days in 12 stroke patients at the subacute stage and demonstrated that hand function was improved, and FA value of the CST in the posterior limb of the internal capsule was increased (Wei et al., 2013). Subsequently, Chen et al. (2014) investigated the effect of NMES for 3 weeks in 48 stroke patients at the early stage, and they found that NMES improved motor function and increment of FA value of the CST around the lesion area. Although results of the above two DTI studies appeared to demonstrate an effect of NMES on the CST using DTI, these studies investigated the effects only in a specific area of the CST pathway. Therefore, to the best of our knowledge, this is the first study to demonstrate an effect of NMES for the entire CST by NMES training on the finger extensor muscles, evaluated by DTT in normal subjects.

However, the limitations of this study should be considered. First, DTT can produce false negative results throughout the white matter of the brain because of crossing fiber or partial volume effect (Parker and Alexander, 2005). Second, we investigated the effect of the NMES training for 2 weeks and could not evaluate the long-term effect of NMES training. Third, there might be a ceiling effect in the evaluation of clinical data of the right hand in the right-handed subjects. As a result, alternative assign of the right hand or the left hand as a training side could have ruled out the possibility of a ceiling effect. Fourth, this study included a small number of subjects. Fifth, we did not rule out the possibility by the sensory stimulation which was applied by electrical current during NMES training in improvement of the hand function. Last, we used the opposite side of the NMES application as the control side instead of recruiting a control group. Two times scanning of DTI was not easy in terms of cost and the compliance of the subjects. In addition, sham stimulation for the control side was not applicable because NMES training is not passive stimulation, but active stimulation. The fact that sham stimulation was not applied for the control side might induce placebo effect. However, we think that the placebo effect could be ruled out to a certain degree because the CST on DTT has anatomical characteristics instead of functional characteristics.

In conclusion, we demonstrated the facilitation of the contralateral CST with improvement of fine motor activity by 2 weeks of NMES training on peripheral muscles in normal subjects. We think our results can be applied to the sports training for normal subjects including athletes and the patients with brain injury to improve the fine motor function of the hand and facilitate the normal subjects or healing of the injured CST. Further long-term follow up studies involving larger numbers of normal subjects and patients with brain injury should be encouraged.

## Author Contributions

SH: study concept and design, manuscript development and writing. YS: study concept and design, acquisition and analysis of data, manuscript authorization.

## Conflict of Interest Statement

The authors declare that the research was conducted in the absence of any commercial or financial relationships that could be construed as a potential conflict of interest.

## References

[B1] Assaf Y. Pasternak O. (2008). Diffusion tensor imaging (DTI)-based white matter mapping in brain research: a review. J. Mol. Neurosci. 34, 51–61. 10.1007/s12031-007-0029-0 18157658

[B2] Brandstack N. Kurki T. Laalo J. Kauko T. Tenovuo O. (2016). Reproducibility of tract-based and region-of-interest DTI analysis of long association tracts. Clinl. Neuroradiol. 26, 199–208. 10.1007/s00062-014-0349-8 25283182

[B3] Chae J. Yu D. (2000). A critical review of neuromuscular electrical stimulation for treatment of motor dysfunction in hemiplegia. Assist. Technol. 12, 33–49. 10.1080/10400435.2000.10132008 11067576

[B4] Chang W. H. Uhm K. E. Shin Y. I. Pascual-Leone A. Kim Y. H. (2016). Factors influencing the response to high-frequency repetitive transcranial magnetic stimulation in patients with subacute stroke. Restor. Neurol. Neurosci. 34, 747–755. 10.3233/rnn-150634 27372515

[B5] Chen D. Yan T. Li G. Li F. Liang Q. (2014). Functional electrical stimulation based on a working pattern influences function of lower extremity in subjects with early stroke and effects on diffusion tensor imaging: a randomized controlled trial. Zhonghua Yi Xue Za Zhi 94, 2886–2892. 10.3760/cma.j.issn.0376-2491.2014.37.003 25549639

[B6] Daly J. J. Ruff R. L. (2007). Construction of efficacious gait and upper limb functional interventions based on brain plasticity evidence and model-based measures for stroke patients. ScientificWorldJournal 7, 2031–2045. 10.1100/tsw.2007.299 18167618 PMC5901328

[B7] Danielian L. E. Iwata N. K. Thomasson D. M. Floeter M. K. (2010). Reliability of fiber tracking measurements in diffusion tensor imaging for longitudinal study. Neuroimage 49, 1572–1580. 10.1016/j.neuroimage.2009.08.062 19744567 PMC2789889

[B8] Davidoff R. A. (1990). The pyramidal tract. Neurology 40, 332–339. 10.1212/WNL.40.2.332 2405296

[B27] de Oliveira Melo M. Aragao F. A. Vaz M. A. (2013). Neuromuscular electrical stimulation for muscle strengthening in elderly with knee osteoarthritis–a systematic review. Complement. Ther. Clin. Pract. 19, 27–31. 10.1016/j.ctcp.2012.09.002 23337561

[B9] Doucet B. M. Lam A. Griffin L. (2012). Neuromuscular electrical stimulation for skeletal muscle function. Yale J. Biol. Med. 85, 201–215. 22737049 PMC3375668

[B10] Han B. S. Jang S. H. Chang Y. M. Byun W. M. Lim S. K. Kang D. S. (2003). Functional magnetic resonance image finding of cortical activation by neuromuscular electrical stimulation on wrist extensor muscles. Am. J. Phys. Med. Rehabil. 82, 17–20. 10.1097/00002060-200301000-00003 12510180

[B11] Jang S. H. (2014). The corticospinal tract from the viewpoint of brain rehabilitation. J. Rehabil. Med. 46, 193–199. 10.2340/16501977-1782 24531325

[B12] Jang S. H. (2016). Dignostic history of traumatic axonal injury in patients with cerebral concussion and mild traumatic brain injury. Brain Neurorehabil. 9, 1–8. 10.12786/bn.2016.9.e1

[B13] Jang S. H. Chang C. H. Lee J. Kim C. S. Seo J. P. Yeo S. S. (2013). Functional role of the corticoreticular pathway in chronic stroke patients. Stroke 44, 1099–1104. 10.1161/strokeaha.111.000269 23444306

[B14] Jang S. H. Jang W. H. Chang P. H. Lee S.-H. Jin S.-H. Kim Y. G.. (2014a). Cortical activation change induced by neuromuscular electrical stimulation during hand movements: a functional NIRS study. J. Neuroeng. Rehabil. 11:29. 10.1186/1743-0003-11-29 24597550 PMC3973889

[B15] Jang S. H. Kim K. Kim S. H. Son S. M. Jang W. H. Kwon H. G. (2014b). The relation between motor function of stroke patients and diffusion tensor imaging findings for the corticospinal tract. Neurosci. Lett. 572, 1–6. 10.1016/j.neulet.2014.04.044 24796808

[B16] Khedr E. M. Etraby A. E. Hemeda M. Nasef A. M. Razek A. A. (2010). Long-term effect of repetitive transcranial magnetic stimulation on motor function recovery after acute ischemic stroke. Acta Neurol. Scand. 121, 30–37. 10.1111/j.1600-0404.2009.01195.x 19678808

[B17] Kim K. M. Croy T. Hertel J. Saliba S. (2010). Effects of neuromuscular electrical stimulation after anterior cruciate ligament reconstruction on quadriceps strength, function and patient-oriented outcomes: a systematic review. J. Orthop. Sports. Phys. Ther. 40, 383–391. 10.2519/jospt.2010.3184 20592480

[B19] Kim Y. T. Kang S. Y. Kim H. S. Shin B. S. (1994). Hand strength and dextricity evaluation with age. J. Kor. Acad. Rehabil. Med. 18, 780–788.

[B18] Kim Y.-H. You S.-H. Ko M.-H. Park J.-W. Lee K. H. Jang S. H.. (2006). Repetitive transcranial magnetic stimulation-induced corticomotor excitability and associated motor skill acquisition in chronic stroke. Stroke 37, 1471–1476. 10.1161/01.str.0000221233.55497.51 16675743

[B20] Kong S. Lee K. S. Kim J. Jang S. H. (2014). The effect of two different hand exercises on grip strength, forearm circumference and vascular maturation in patients who underwent arteriovenous fistula surgery. Ann. Rehabil. Med. 38, 648–657. 10.5535/arm.2014.38.5.648 25379494 PMC4221393

[B21] Kunimatsu A. Aoki S. Masutani Y. Abe O. Hayashi N. Mori H.. (2004). The optimal trackability threshold of fractional anisotropy for diffusion tensor tractography of the corticospinal tract. Magn. Reson. Med. Sci. 3, 11–17. 10.2463/mrms.3.11 16093615

[B22] Lee H. D. Jang S. H. (2015). Injury of the corticoreticular pathway in patients with mild traumatic brain injury: a diffusion tensor tractography study. Brain Inj. [Epub ahead of print]. 10.3109/02699052.2015.1045028 26204321

[B23] Maddocks M. Gao W. Higginson I. J. Wilcock A. (2013). Neuromuscular electrical stimulation for muscle weakness in adults with advanced disease. Cochrane Database Syst. Rev. 1:CD009419. 10.1002/14651858.CD009419.pub2 23440837

[B24] Malykhin N. Concha L. Seres P. Beaulieu C. Coupland N. J. (2008). Diffusion tensor imaging tractography and reliability analysis for limbic and paralimbic white matter tracts. Psychiatry Res. 164, 132–142. 10.1016/j.pscychresns.2007.11.007 18945599

[B25] Mang C. S. Clair J. M. Collins D. F. (2011). Neuromuscular electrical stimulation has a global effect on corticospinal excitability for leg muscles and a focused effect for hand muscles. Exp. Brain Res. 209, 355–363. 10.1007/s00221-011-2556-8 21286692

[B26] Mang C. S. Lagerquist O. Collins D. F. (2010). Changes in corticospinal excitability evoked by common peroneal nerve stimulation depend on stimulation frequency. Exp. Brain Res. 203, 11–20. 10.1007/s00221-010-2202-x 20217400

[B28] Mori S. Crain B. J. Chacko V. P. van Zijl P. C. (1999). Three-dimensional tracking of axonal projections in the brain by magnetic resonance imaging. Ann. Neurol. 45, 265–269. 10.1002/1531-8249(199902)45:2<265::aid-ana21>3.0.co;2-3 9989633

[B29] Nakipğlu Yuzer G. F. Köse dönmez B. Ozgirgin N. (2017). A randomized controlled study: effectiveness of functional electrical stimulation on wrist and finger flexor spasticity in hemiplegia. J. Stroke Cerebrovasc. Dis. 26, 1467–1471. 10.1016/j.jstrokecerebrovasdis.2017.03.011 28462794

[B30] Neil J. J. (2008). Diffusion imaging concepts for clinicians. J. Magn. Reson. Imaging 27, 1–7. 10.1002/jmri.21087 18050325

[B31] Oldfield R. C. (1971). The assessment and analysis of handedness: the edinburgh inventory. Neuropsychologia 9, 97–113. 10.1016/0028-3932(71)90067-4 5146491

[B32] Pagani E. Agosta F. Rocca M. A. Caputo D. Filippi M. (2008). Voxel-based analysis derived from fractional anisotropy images of white matter volume changes with aging. Neuroimage 41, 657–667. 10.1016/j.neuroimage.2008.03.021 18442927

[B33] Parker G. J. M. Alexander D. C. (2005). Probabilistic anatomical connectivity derived from the microscopic persistent angular structure of cerebral tissue. Philos. Trans. R. Soc. Lond. B Biol. Sci. 360, 893–902. 10.1098/rstb.2005.1639 16087434 PMC1854923

[B34] Powell J. Pandyan A. D. Granat M. Cameron M. Stott D. J. (1999). Electrical stimulation of wrist extensors in poststroke hemiplegia. Stroke 30, 1384–1389. 10.1161/01.str.30.7.1384 10390311

[B35] Puig J. Pedraza S. Blasco G. Daunis-i-Estadella A. Prats A. Prados F.. (2010). Wallerian degeneration in the corticospinal tract evaluated by diffusion tensor imaging correlates with motor deficit 30 days after middle cerebral artery ischemic stroke. Am. J. Neuroradiol. 31, 1324–1330. 10.3174/ajnr.a2038 20299434 PMC7965455

[B36] Pundik S. McCabe J. Skelly M. Tatsuoka C. Daly J. J. (2018). Association of spasticity and motor dysfunction in chronic stroke. Ann. Phys. Rehabil. Med. [Epub ahead of print]. 10.1016/j.rehab.2018.07.006 30099149

[B37] Reddon J. R. Gill D. M. Gauk S. E. Maerz M. D. (1988). Purdue pegboard: test-retest estimates. Percept. Mot. Skills 66, 503–506. 10.2466/pms.1988.66.2.503 3399326

[B38] Rushton D. N. (1997). Functional electrical stimulation. Physiol. Meas. 18, 241–275. 10.1088/0967-3334/18/4/001 9413861

[B39] Schaechter J. D. Fricker Z. P. Perdue K. L. Helmer K. G. Vangel M. G. Greve D. N.. (2009). Microstructural status of ipsilesional and contralesional corticospinal tract correlates with motor skill in chronic stroke patients. Hum. Brain Mapp. 30, 3461–3474. 10.1002/hbm.20770 19370766 PMC2780023

[B40] Seo J. P. Jang S. H. (2014). Injury of the spinothalamic tract in a patient with mild traumatic brain injury: diffusion tensor tractography study. J. Rehabil. Med. 46, 374–377. 10.2340/16501977-1783 24577424

[B41] Seo J. P. Jang S. H. (2015). Traumatic axonal injury of the corticospinal tract in the subcortical white matter in patients with mild traumatic brain injury. Brain Inj. 29, 110–114. 10.3109/02699052.2014.973447 25356741

[B42] Shin H. K. Cho S. H. Jeon H.-S. Lee Y. H. Song J. C. Jang S. H.. (2008). Cortical effect and functional recovery by the electromyography-triggered neuromuscular stimulation in chronic stroke patients. Neurosci. Lett. 442, 174–179. 10.1016/j.neulet.2008.07.026 18644424

[B43] Smith R. O. Benge M. W. (1985). Pinch and grasp strength: standardization of terminology and protocol. Am. J. Occup. Ther. 39, 531–535. 10.5014/ajot.39.8.531 4037051

[B44] Tiffin J. Asher E. J. (1948). The purdue pegboard; norms and studies of reliability and validity. J. Appl. Psychol. 32, 234–247. 10.1037/h0061266 18867059

[B45] Wakana S. Caprihan A. Panzenboeck M. M. Fallon J. H. Perry M. Gollub R. L.. (2007). Reproducibility of quantitative tractography methods applied to cerebral white matter. Neuroimage 36, 630–644. 10.1016/j.neuroimage.2007.02.049 17481925 PMC2350213

[B46] Wang J. Y. Abdi H. Bakhadirov K. Diaz-Arrastia R. Devous M. D. (2012). A comprehensive reliability assessment of quantitative diffusion tensor tractography. Neuroimage 60, 1127–1138. 10.1016/j.neuroimage.2011.12.062 22227883 PMC3468740

[B47] Wei W. J. Bai L. J. Wang J. Dai R. W. Tong R. K. Y. Zhang Y. M.. (2013). A longitudinal study of h and motor recovery after sub-acute stroke: a study combined fMRI with diffusion tensor imaging. PLOs One 8:e64154. 10.1371/journal.pone.0064154 23724030 PMC3665895

[B48] Weinstein M. Myers V. Green D. Schertz M. Shiran S. I. Geva R.. (2015). Brain plasticity following intensive bimanual therapy in children with hemiparesis: preliminary evidence. Neural Plast. 2015:798481. 10.1155/2015/798481 26640717 PMC4657087

[B49] Yamada K. Mori S. Nakamura H. Ito H. Kizu O. Shiga K.. (2003). Fiber-tracking method reveals sensorimotor pathway involvement in stroke patients. Stroke 34, E159–E162. 10.1161/01.str.0000085827.54986.89 12907811

[B50] York D. H. (1987). Review of descending motor pathways involved with transcranial stimulation. Neurosurgery 20, 70–73. 10.1097/00006123-198701000-00021 3543726

